# Comparison of Morphometric Aspects of Light and Electron Microscopy of the Hypoglossal Nerve between Young and Aged Male Wistar Rats

**Published:** 2011-12-22

**Authors:** Nabiollah Soltanpour, Yasser Asghari Vostacolaee, Mohsen Pourghasem

**Affiliations:** Department of Anatomy, Biology and Molecular Research Center , Babol University of Medical Sciences, Babol, Iran

**Keywords:** Hypoglossal Nerve, Myelinated Nerve Fiber, Aging, Rat

## Abstract

**Objective:**

Age-related changes occur in many different systems of the body. Many elderly people show dysphagia and dysphonia. This research was conducted to evaluate quantitatively the morphometrical changes of the hypoglossal nerve resulting from the aging process in young and aged rats.

**Materials and Methods:**

Through an experimental study ten male wistar rats (4 months: 5 rats, 24 months: 5 rats) were selected randomly from a colony of wistars in the UWC. After a fixation process and preparation of samples of the cervical portion of the hypoglossal nerve of these rats, light and electron microscopic imaging were performed. These images were evaluated according to the numbers and size of myelinated nerve fibers, nucleoli of Schwann cells, myelin sheath thickness, axon diameter, and g ratio. All data were analyzed by Mann-Whitney, a non-parametric statistical test.

**Results:**

In light microscope, numbers of myelinated nerve fibers, the mean entire nerve perimeters, the mean entire nerve areas and the mean entire nerve diameters in young and aged rats' were not significantly different between the two groups.

In electron microscope, numbers of myelinated axons, numbers of Schwann cell nucleoli and the mean g ratios of myelinated axon to Schwann cell in young and aged rats were not significantly different. The myelinated fiber diameters, the myelin sheath thicknesses, myelinated axon diameters and the mean g ratio of axon diameter to myelinated fiber diameter in young and aged fibers were significantly different

**Conclusion:**

The mean g ratio of myelinated nerve fibers of peripheral nerves stabilizes at the level of 0.6 after maturation and persists without major change during adulthood. This ratio of axon diameter to fiber diameter (0.6) is optimum for normal conduction velocity of neural impulses. Our study indicated that the g ratio of myelinated nerve fiber of the hypoglossal nerve decreased prominently in aged rats and can be a cause of impairment in nerve function in old age. Thus, prospective studies concerning electrophysiological and conductive properties of the peripheral nerve could be useful to clarify further the effects of aging on peripheral nerves.

## introduction

The hypoglossal nerve contains motor neurons of the tongue. There is a limited number of studies on aging-related changes in the morphological composition of the cranial nerves, mainly the hypoglossal nerve, in different species, in comparison to somatic peripheral nerves, perhaps on account of the relative ease and practicality of obtaining biopsies of the latter (1), such as the sural nerve (2, 3). Peripheral nerve function is significantly affected by maturation and aging (4-6). The aging process triggers modifications in the human body that are responsible for many different types of clinical manifestations, represented in the upper aero digestive tract as vocal disorders and swallowing disorders (7).

Oropharyngeal dysphagia is a frequent symptom in the elderly, especially in men aged over 60, and it is normally associated with an increase in the duration of the oropharyngeal phase of swallowing (8). Many different authors have demonstrated that the aging process is also related to reduction of pharyngeal and supraglotic sensitivity and is considered a factor responsible for the onset of dysphagia, aspiration and repetitive pneumonia in the elderly, owing to reduction of reflexes that protect the lower airway (9). A study on tongue muscle contractile power demonstrated that the aging process affects protrusive contraction of the tongue muscles in rats and so decreases tetanic forces of the tongue. These changes are similar to human models and may be associated with age-related changes in the swallowing function (10). So, it is important that information on the hypoglossal nerve's morphology and axonal morphometric parameters in older age is available in order to determine whether there may be a peripheral contribution to age-associated changes in motor function of the tongue, pharynx and upper aerodigestive tract. The present morphometric study of the hypoglossal nerve has been undertaken in order to investigate whether morphological changes occur in this nerve in rats.

## Materials and Methods

### Tissue preparation

This research is approved by the Ethical Committee of Cardiff University. Male white wistar rats, from a colony of wistars at the University of Wales, Cardiff (UK), kept under constant conditions of temperature and humidity, fed water and chow ad libitum, and maintained under barrier conditions, were used in this study (11). Animals of two age groups: 4- and 24- months (n=5 per group) were perfused via the left ventricle under ether anaesthesia as follows: perfusion with 200 ml of phosphate buffered saline containing heparin (25 units/ml) at 37℃ for eight minutes was followed by perfusion with 3% glutaraldehyde in 100 mM sodium cacodylate buffer, pH= 7.3 at 4℃ for 30 minutes. The left and right hypoglossal nerves were sectioned at the upper cervical level, then diced into small pieces and transferred to fresh fixative for two hours at 4℃. After rinsing in 100 mM sodium cacodylate buffer, osmication in 1% osmium tetroxide in cacodylate buffer and dehydration, the tissue was embedded in Spurr's resin. For electron microscopy, ultrathin sections were cut on a Reichert Ultrcut, stained with lead citrate and uranyl acetate and examined with a Phillips 400 electron microscope. For light microscopy, the blocks were sectioned at 2 µm with every tenth section being saved and mounted in order on glass slides. Each set of sections was stained with 1% toluidine blue.

### Morphometry

Photomicrographs of the 2µm sections were printed to make montages of the entire nerve at a final magnification of ×220. Ten montages, five from each side, were made of each two age groups. The cross-sectional areas and perimeters of the entire sections of the hypoglossal nerves of both age groups were measured by a computerized tissue-image analyzer software called Motic Image Plus; 2005. Numbers of the myelinated nerve fibers were counted manually (11).

For electron microscopy, electron micrographs of six areas from each five hypoglossal nerve -- a total of 30 areas from each two age groups (sampled by a standardized random protocol designed to give every part of the nerve an equal chance of being sampled) -- were taken at ×1500 and printed at a final magnification of ×5250. This represented an area of 2465µm^2^.

The total numbers of myelinated fibers were counted in each micrograph. In this protocol the myelinated fibers overlying the upper and left margins were included in the counts whereas those overlying the lower and right margins were excluded. Myelinated fiber/axon diameters were determined by measurement of their smallest diameters. Myelin sheath thickness was determined using a radiating set of straight lines separated by 60° on a myelin sheath, overly superimposed on the centre of each myelinated axon with the result that the lines transected the myelin sheath at six points. At the points of intersection of each line with the innermost lamella of the sheath, the thickness was measured perpendicular to that point manually, and averaged. Axonal diameters were calculated by subtracting ×2 the mean myelin sheath thickness from the measured ‘fiber’ diameter. Ideally the myelin sheath thickness measurement should be made at the point showing the smallest sheath thickness but in older age groups the inner and outer lamellae of the myelin sheaths were not always as distinct as in the young group. The ratio of the axon diameter to the total fiber diameter (g ratio) was also calculated. The numbers of axons measured were a mean of 1000 for each group. All data were analyzed by Mann-Whitney a non-parametric statistical test and a p <0.05 was considered significant.

**Table 1:** Counts obtained from light microscopic montages of transverse sections of the cervical hypoglossal nerve


Age(months)	4	24	P-value
Total numbers of myelinated fiber	3357.5 ± 406.18	3272.4 ± 396.69	0.572
Entire nerve perimeter; transverse section(mm)	1.559 ± 0.288	1.507 ± 0.194	0.787
Entire nerve area; transverse section(mm^2^)	0.188 ± 0.071	0.181 ± 0.036	0.880
Entire nerve diameter; transverse section(mm)	0.450 ± 0.129	0.435 ± 0.087	0.726


Note: p < 0.05 is significant.

## Results

### A Light microscopy

**Fig 1:**
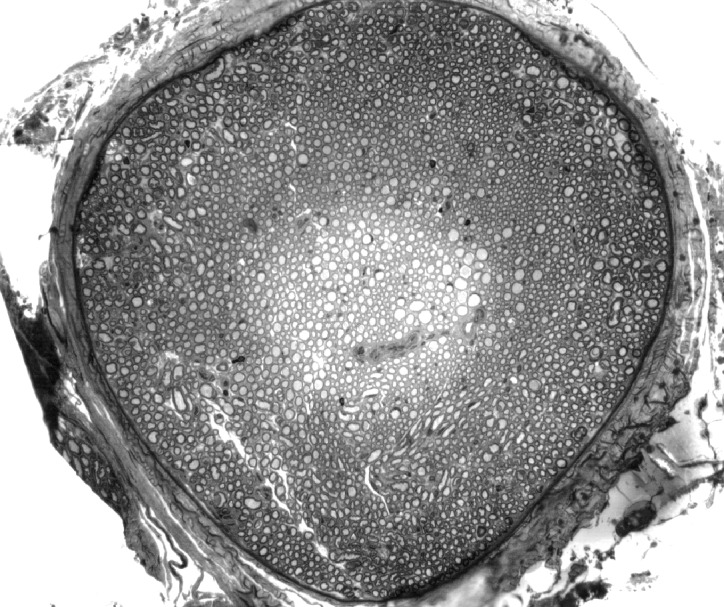
Representative light microscopic transverse section of the 4-month old hypoglossal nerve (×132).Stained with Toluidine blue.

The cervical hypoglossal nerve trunk was enclosed within a collagenous epineurium but not subdivided into fascicles (Fig 1). The mean values for the entire nerve cross-sectional diameter, perimeter, and area and the total numbers of myelinated fibers are given in table 1. No differences were observed in comparison between groups. Although total numbers of myelinated fibers decreased with age, difference was not statistically considerable (p=0.572). There were also slight but not a significant decreases in the entire nerve cross-sectional diameter (p=0.726), perimeter (p= 0.787) and area (p= 0.880) with aging (Table 1).

### Electron microscopy-1

**Fig 2:**
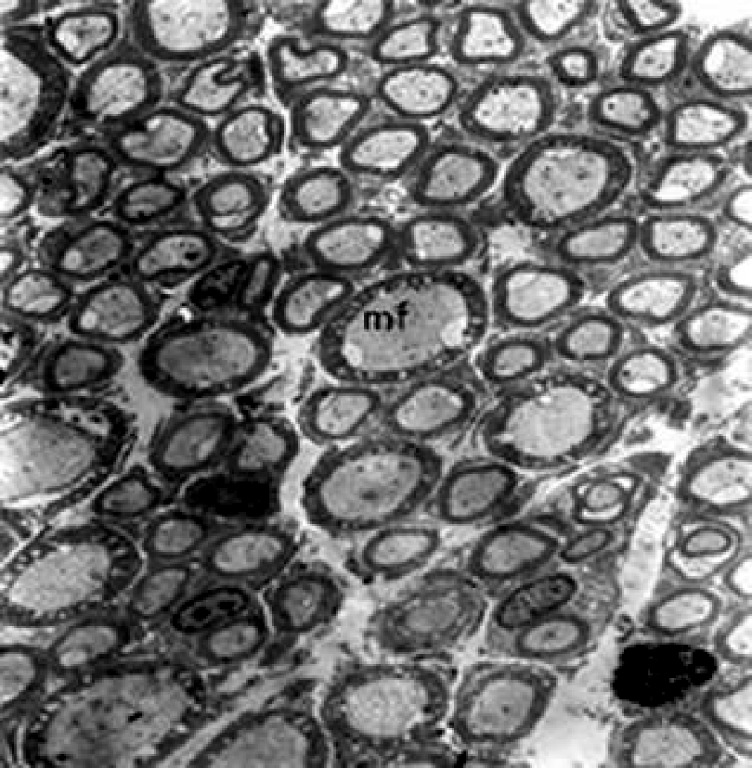
Representative electron micrograph from a transverse section of the 4-month-old hypoglossal nerve (×2625). mf; Myelinated fiber; mf

The mean values for the numbers of myelinated axons, numbers of Schwann cell nucleoli and ratio of myelinated axon to Schwann cell nucleous per mm^2^ (Fig 2) are given in table 2. Although these parameters decreased in 24-month-old rats compared with 4-month-old rats, there were no significant changes with aging (p>0.05).

**Table 2:** Counts obtained from electron microscopic micrographs; per 1 mm^2^


Age(months)	4	24	P-value
Numbers of myelinated axons	22888.49 ± 7776.88	21906.72 ± 108.94	0.367
Numbers of Schwann cell nucleoli	957.40 ± 685.59	957.40 ± 827.58	0.147
Ratio of myelinated axon to Schwann cell	11736.32 ± 6227.18	11314.41 ± 668.60	0.74


Note: p < 0.05 is significant.

**Table 3:** Myelinated fiber measurements from electron micrographs of the cervical hypoglossal nerve


Age(months)	4	24	P-value
Myelinated fiber diameter (µm)	3.7 ± 1.34	3.24 ± 1.55	0.005
Myelin sheath thickness (µm)	0.69 ± 0.27	0.73 ± 0.33	0.002
Myelinated axon diameter (µm)	2.37 ± 0.98	1.77 ± 1.05	0.005
g ratio	0.62 ± 0.096	0.52 ± 0.12	0.000


Note: p < 0.05 is significant.

### Electron microscopy-2

The mean values for myelinated fiber diameter, myelin sheath thickness, myelinated axon diameter and g ratio of axon diameter to myelinated fiber diameter as shown in table 3, decreased significantly (p< 0.05) with aging.

**Fig 3:**
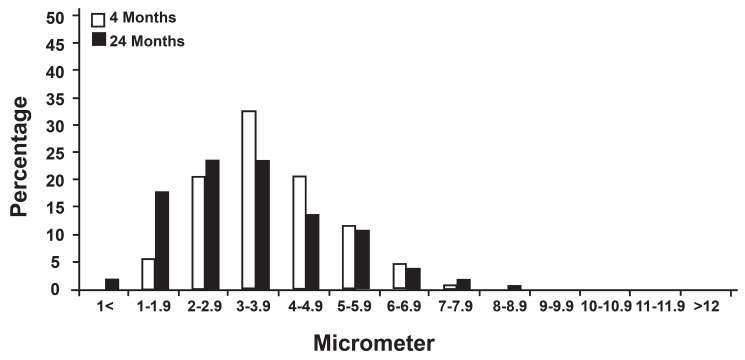
Distribution of myelinated fiber diameter in electron micrographs of the hypoglossal nerve.

**Fig 4:**
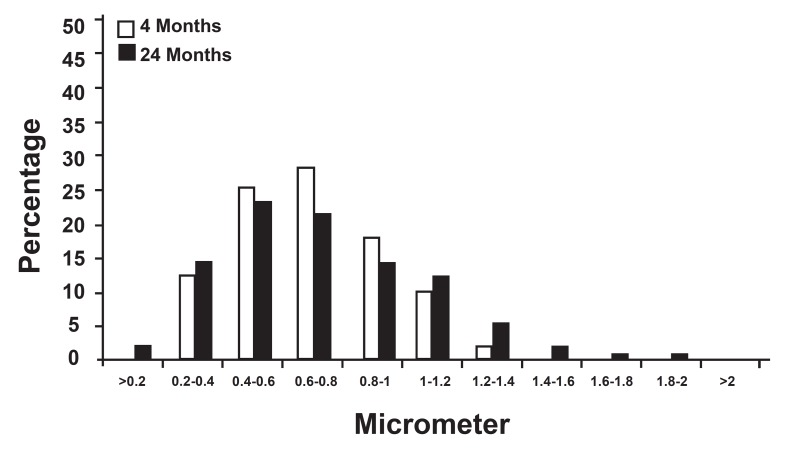
Distribution of myelin sheath thickness in electron micrographs of the hypoglossal nerve

**Fig 5:**
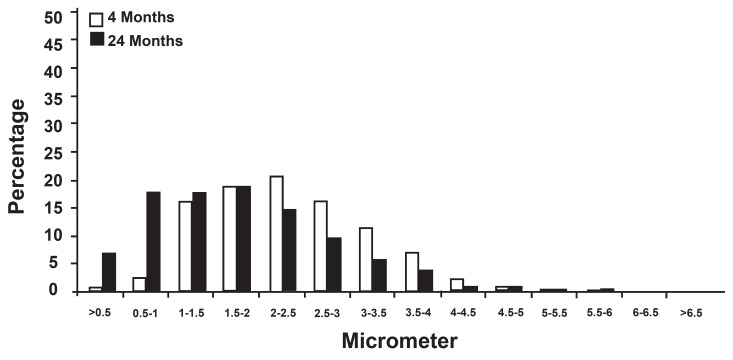
Distribution of axon diameter in electron micrographs of the hypoglossal nerve.

**Fig 6:**
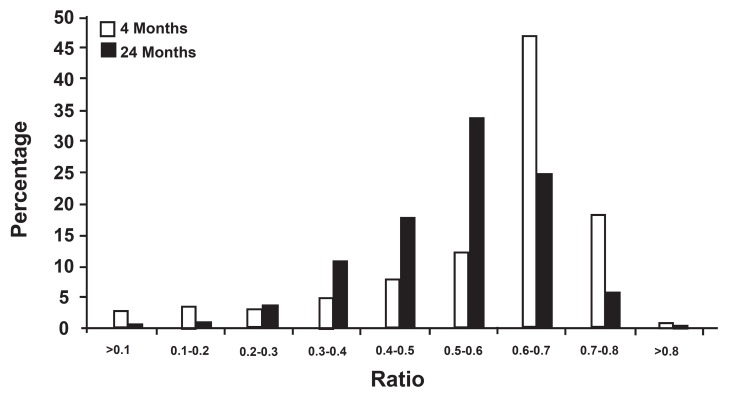
Distribution of g ratio in electron micrographs of the hypoglossal nerve.

The distributions of measurements made on the nerve fiber populations are presented in figures 3-6.

In the two age groups, the measurement of the myelinated fiber diameter did not present a similar unimodel distribution (the majority of the fibers had a 2 to 5 µm diameter in the 4-month group but 1 to 4 µm diameter in the 24-month group), and all showed significant decrease between 4 and 24 months (p<0.05).

The distribution of myelin sheath thickness, myelinated axon diameter and g ratio distribution were all unimodal but not similar in myelinated axon diameter and g ratio in both age groups (majority of the axons had a 1 to 3 µm diameter in the 4-month group, but 0.5 to 2.5 µm diameter in the 24-month group and the majority of the g ratios were 0.6 to 0.7 at 4 months, but 0.5 to 0.6 at 24 months ).

## Discussion

There are many studies in literature about agerelated changes in the structure and function of the nervous system. But this study provided more extensive data about age-related morphometric changes in the cervical hypoglossal nerve of rats. 

As previously mentioned, peripheral nerve function is significantly affected by maturation and aging (4). Rats are less susceptible to spontaneously peripheral neuropathy (12), so we selected them for the present study.

The present study showed that in rats cross-sectional perimeter, area and diameter and total numbers of myelinated fibers change slightly but not to a statistically significant extent (p>0.05). This finding is compatible with other results (13, 14). In a previous study we showed that morphology of the cervical vagus nerve of the rat is maintained without overt deterioration throughout the adult lifespan. Although decreases in myelinated and unmyelinated nerve numbers calculated from electron micrographs were statistically significant, they were in fact only small changes (15).

A study on tongue contractile power showed a decrease in tongue muscle protrusive forces (10) and another study demonstrated an increase in the duration of the oropharyngeal phase of swallowing (8) through aging, which causes dysphagia, aspiration and other clinical manifestations of the upper aerodigestive tract in the elderly (9).Rats have a short lifespan compared with humans. This could explain why minimal alterations have been seen in our study.

Jeronimo et al. in a study on the rat's sural nerve, showed that in rats there is a postnatal growth spurt between the first and third months of life (30 and 90 days), as judged by increases in body weight, which is associated with changes in myelinated fiber diameter in peripheral nerves. His study also indicated that fiber population distribution changes with increasing body weight (4). This finding is compatible with the result obtained by Jacobs and Love in human sural nerves (16). Body weight continued to increase at a less rapid rate up to the age of six months (180 days) but nerve parameters tended to stabilize between the ages of three and six months (90 and 180 days) (4). This is also in agreement with studies showing that morphometric parameters of nerve fibers are stable from six months to older ages (11).

Fascicle cross-sectional area of sural nerve, in Jeronimo et al. study, showed a significant increase in three and six month old rats compared with one month old rats. In contrast, myelinated fiber density decreased significantly in three and six month old rats compared with one month old rats, but there was no significant difference between three and six month old rats (4). Although according to the present study cross-sectional area and perimeter of the hypoglossal nerve did not change significantly after maturation and during aging up to 24 months, slight changes in these parameters may be causes of some considerable changes after 24 months.

In the study of Jeronimo et al. the increase in the fascicle area observed from one to three months was largely related to the increase in myelinated fiber size; by contrast from three to six months, this increase was related to an increase in the connective tissue components (4). Although in our study the myelinated fiber diameter of the hypoglossal nerve decreased significantly through aging up to 24 months, there was no significant change in the cross-sectional area of the nerve. The amount of endoneurial connective tissue increases with aging and this causes the cross-sectional area of the hypoglossal nerve to be constant during aging (4, 16, 17).

Jeronimo et al. demonstrated that total number of myelinated fibers and Schwann cell nucleoli are similar on both sides and age groups of premature and matured sural nerves. We found that there were slight but not significant decreases in total numbers of myelinated fiber and Schwann cell nucleous after maturation, during aging up to 24 months. It can be hypothesized that these changes may cause more considerable changes in older ages, after 24 months.

According to the present study, a significant decrease in g ratio can cause dysfunction in nerve impulse conduction in aged rats, so it is necessary to design another study to investigate age-related changing of electrophysiological properties and nerve conduction velocity of the hypoglossal nerve. In the present study the g ratio in myelinated fibers of the hypoglossal nerve reached 0.6 at the age of four months. This is quite similar to our previous study in which we demonstrated that the vagus nerve of the male wistar rat reached0.6 in g ratio of myelinated fibers at four months of age (11). But in Jeronimo et al. study, it was shown that the g ratio of myelinated fibers of the sural nerve reach to 0.6 at the age of 6 months (4).

So it can be hypothesized that the peripheral nerves originating from the brain, such as the hypoglossal nerve, have earlier maturation compared with the peripheral nerves originating from the spinal cord, such as sural nerves

Correlation between the myelin sheath and diameter of the respective axon has been known since 1905 (18) and may differ significantly between nerves and also between large and small fiber classes within individual nerves (19). Our results are in accordance with those describing a thicker myelin sheath in large axons (19- 21).

## Conclusion

The results of the present study showed that the gross morphometric aspects (light microscopy) of the cervical hypoglossal nerve of the rat are maintained without overt change throughout aging. Although the decreases in myelinated nerve numbers and g ratios measured from electron micrographs are statistically significant, they may in fact be small changes, so more electrophysiological and nerve function studies are needed to prove it.
